# Simethicone administration improves gastric cleanness for esophagogastroduodenoscopy: a randomized clinical trial

**DOI:** 10.1186/s13063-021-05527-8

**Published:** 2021-08-21

**Authors:** Xiaotian Sun, Yang Xu, Xueting Zhang, Cuiyun Ma, Aitong Li, Haiyan Yu, Weihua Zhang, Hanqing Zhang, Teng Yang, Xinfang Miao, Huiming Zhang, Yan Liu, Zheng Lu

**Affiliations:** 1 Gastrointestinal Endoscopy Center, The Fifth Medical Center of Chinese PLA General Hospital, Beijing, 100071 China; 2 Department of Internal Medicine, Beijing South Medical District, Chinese PLA General Hospital, Beijing, 100161 China; 3 Clinic of Fuxing Road, Beijing South Medical District, Chinese PLA General Hospital, Beijing, 100161 China; 4 Department of Hepatology, The Fifth Medical Center of Chinese PLA General Hospital, No. 100 Middle Road in Fourth West Ring, Beijing, 100039 China

**Keywords:** Esophagogastroduodenoscopy, Stomach, Simethicone, Diagnosis

## Abstract

**Background:**

Esophagogastroduodenoscopy is very useful in diagnosing and treating upper gastrointestinal mucosal disorders, but too much foam and water in stomach decrease its diagnostic efficiency. Simethicone administration can help remove excessive foam.

**Aims:**

To determine the optimal simethicone administration strategies in a comparative randomized controlled clinical trial.

**Methods:**

Adult outpatients with indications for esophagogastroduodenoscopy were enrolled and randomly divided into group 1 (simethicone solution intake 20–30 min before procedure, *n* = 110), group 2 (simethicone solution intake 31–60 min before procedure, *n* = 92), and group 3 (simethicone solution intake > 60 min before procedure). Primary and secondary outcomes were procedure time and the patients’ satisfaction after the examination. All symptoms like abdominal pain and distension were recorded.

**Results:**

No statistically significant differences were found on the patients’ demographic and clinical features and mean examination time (all *P* values > 0.05). The distribution of patients with different endoscopic and pathological diagnosis was comparable among three groups, respectively (*P* = 0.607; *P* = 0.289). However, the proportion of patients with Gastric Cleanness Grade A was most in group 2 (*n* = 73, 79.3%), and patient proportion with Gastric Cleanness Grade C was most found in group 1 (*n* = 72, 65.5%), which was greatly different (*P* < 0.001). There was no statistically significant difference on the satisfaction scores [immediately 6 (3–8) vs. 6 (1–10) vs. 6 (1-9), *P* = 0.533; 2 h after 10 (8–10) vs. 10 (10–10) vs. 10 (8-10), *P* = 0.463].

**Conclusion:**

Simethicone solution intake 31–60 min before esophagogastroduodenoscopy can help obtain the best gastric cleanness, which is recommended in clinical practice (registered at ClinicalTrials.gov, NCT03776916 on December 13, 2018).

## What is known?

The presence of too much foam and water in the stomach limited esophagogastroduodenoscopy’s value in managing upper gastrointestinal mucosal disorders. Simethicone can remove excessive foam, while its optimal administration strategy has not been examined.

## What is new here?

Simethicone solution intake 31-60 min before esophagogastroduodenoscopy can effectively help obtain the best gastric cleanness for upper gastrointestinal endoscopy, which can be recommended as a routine in clinical practice.

## Introduction

Esophagogastroduodenoscopy is well acknowledged as the most useful tool for diagnosing and treating upper gastrointestinal tract mucosal lesions [1, 2]. It has the advantage of directly observing the esophageal, gastric, and duodenal mucosa and obtaining biopsy of the potential lesions for pathological examination, thus being widely applied in clinical practice all over the world [3–5]. However, during the examination, too much water, mucus, foam, or residues in the stomach will not only increase the procedure time and the misdiagnosis rate, but also decrease the patients’ tolerance, so more efforts should be made to avoid excessive water, mucus, foam or residues in order to get a clear view of the upper gastrointestinal tract mucosa.

Simethicone is also called poly-dimethylsiloxane, which has been introduced to remove the foam and water. Recent studies have reported that the administration of simethicone before endoscopic examination could shorten the procedure time and improve the diagnostic rate of the gastric mucosal lesions [6, 7]. Although simethicone has been routinely administrated before the esophagogastroduodenoscopy, the optimal strategy of administrating simethicone has not been clearly investigated, especially the time interval from simethicone administration to the endoscopic procedure. Too late intake of simethicone will result in too excessive water in the stomach due to the insufficient time for the stomach to empty, while if the patients take it too early, it does not take effects. Thus, we conducted this randomized controlled clinical trial aiming to optimize the current simethicone administration strategies, and these results could improve the performance of the esophagogastroduodenoscopy and minimize the patients’ dissatisfaction. Different simethicone administration strategies for esophagogastroduodenoscopy were compared and whether the time of taking simethicone before endoscopy could influence the efficacy and efficiency of the endoscopic examination was tested, which may benefit the identification of a standardized protocol for endoscopic procedures.

## Patients and methods

### Patients

Adult outpatients with the indications for esophagogastroduodenoscopy who agreed to participate in the study in Endoscopy Center of the Fifth Medical Center of Chinese PLA General Hospital from December 17, 2018, to March 17, 2019, were included. Patients who were receiving nonsteroidal anti-inflammatory drugs, pump inhibitors (PPI), or antibiotics in the last 3 weeks or had severe uncontrolled coagulopathy, prior history of gastric surgery, or were pregnant and lactation were excluded. All the patients signed written informed consent, which was obtained by the main researcher. The main researcher enrolled the participants and assigned the interventions.

### Study design and grouping

**Fig. 1:**
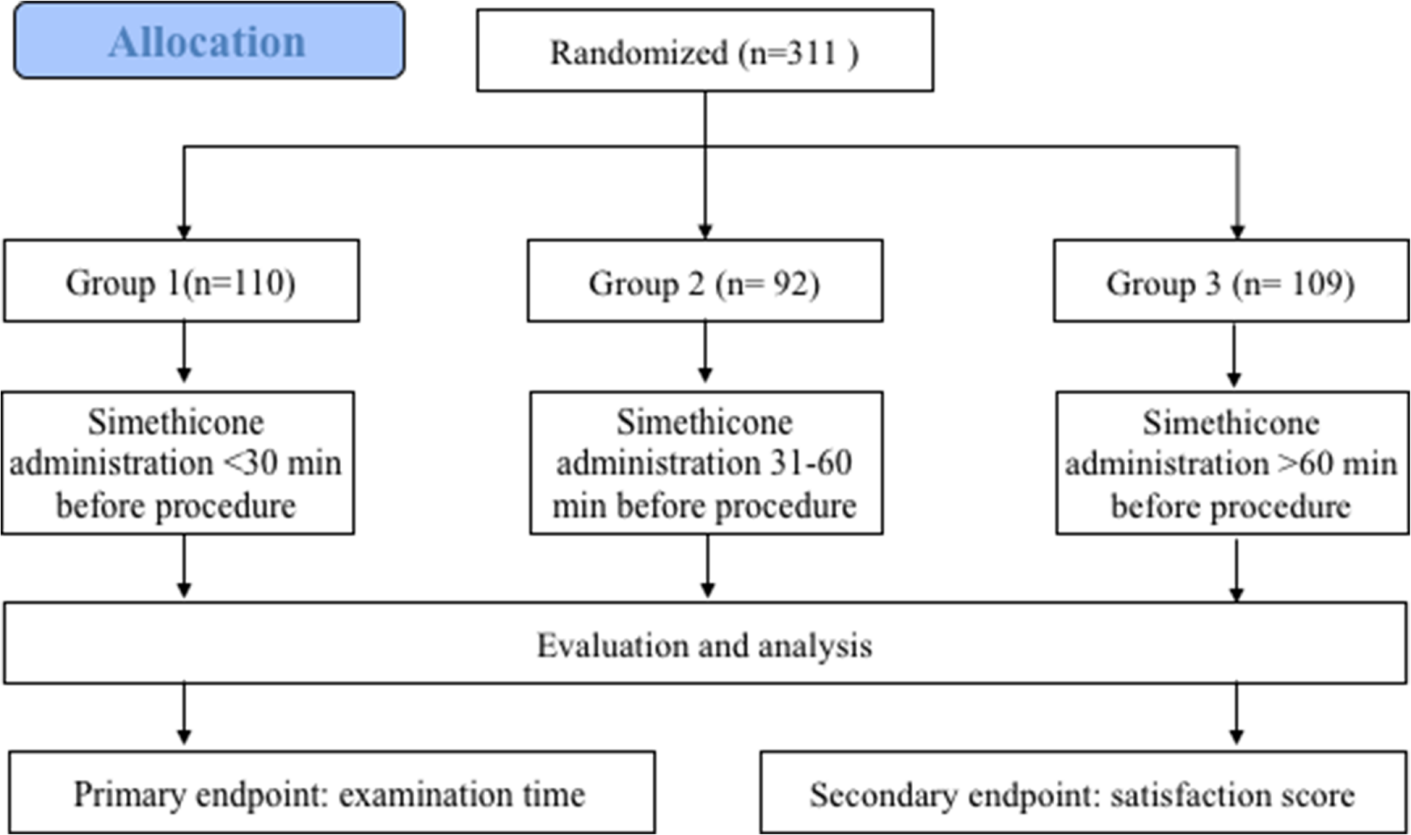
Flow chart of this study

The flow chart was shown in Fig. 1. This study was registered at ClinicalTrials.gov on December 13, 2018, and the registration number was NCT03776916 (https://www.clinicaltrials.gov/ct2/show/NCT03776916?term=NCT03776916&draw=1&rank=1). The protocol was approved by the ethic committee of Affiliated Hospital to Chinese Academy of Military Medical Sciences (the Fifth Medical Center of Chinese PLA General Hospital) on October 12, 2018.

The sample size was calculated to reveal a difference on the gastric cleanness among different groups, keeping a power of 0.9 and significance level alpha of 0.05. Considering that the dropout rate was around 20%, the sample size was estimated to be 300. All the patients were randomly divided into three experimental groups using random number method: group 1 (simethicone solution intake 20–30 min before the procedure, *n* = 110), group 2 (simethicone solution intake 31–60 min before the procedure, *n* = 92), and group 3 (simethicone solution intake > 60 min before the procedure, *n* = 109). An independent researcher who was not involved in the study generated the random allocation sequence, which was stored in sealed envelopes. The envelopes were opened only the randomization was conducted. An independent researcher assistant was responsible for the data collection and management, and all the data were categorized and analyzed based on an electronic datasheet. Another senior researcher was responsible for monitoring and auditing the study. The patients and endoscopists were blinded to the grouping.

### Esophagogastroduodenoscopy

**Fig. 2:**
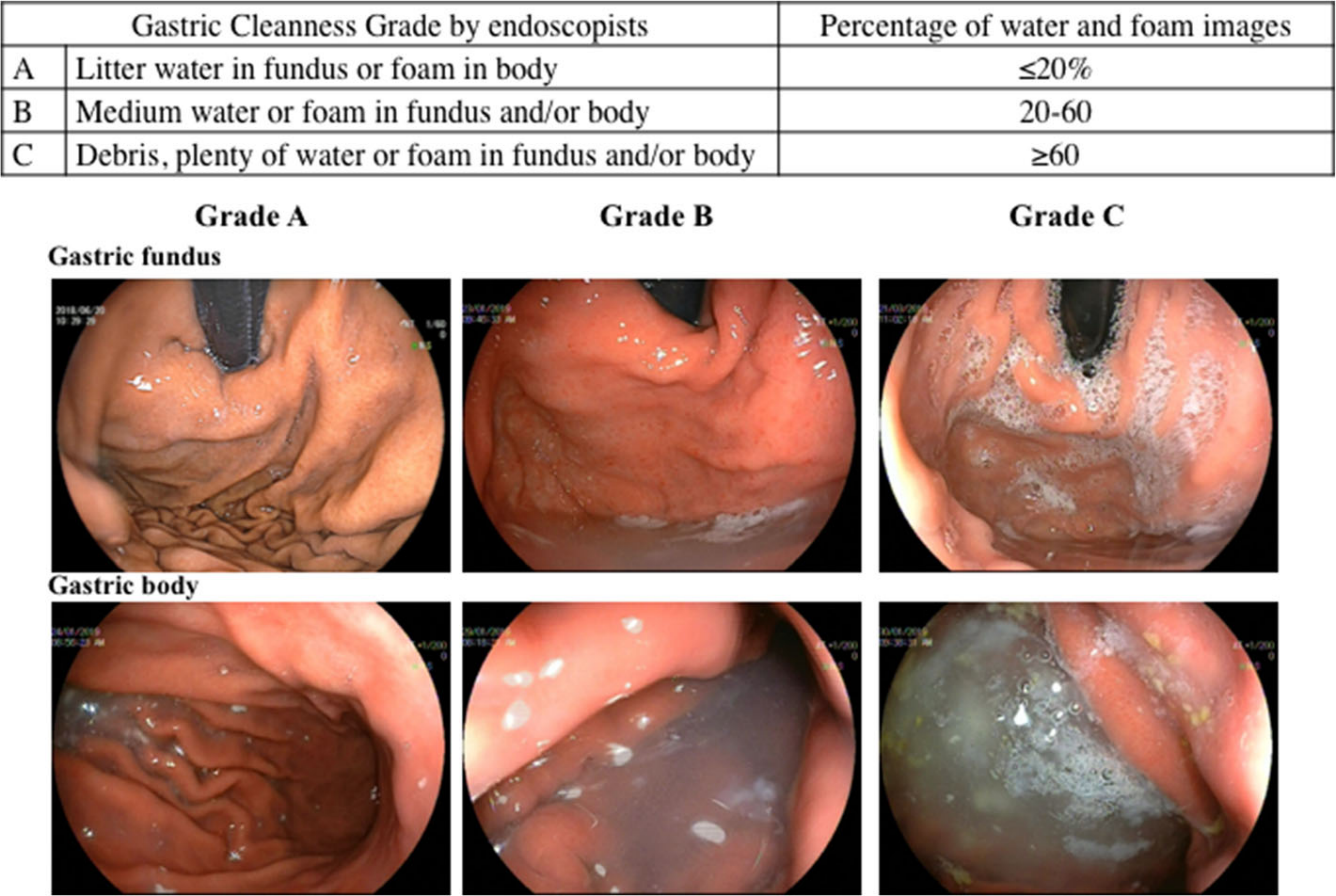
Typical images of the stomach

All endoscopic procedures were completed by one experienced expert endoscopist who had an endoscopy experience of over 5 years. No patients underwent propofol sedation. EG-L590WR endoscopes equipped with the LASEREO endoscopic system (FUJIFILM Co., Tokyo, Japan) were used. The gastric cleanness was evaluated by the endoscopist as Gastric Cleanness Grade and categorized into 3 grades based on the findings of the stomach (Fig. 2). All the endoscopic images taken during the esophagogastroduodenoscopy were evaluated, and the percentage of water and foam in each image was assessed as a subjective evaluator for Gastric Cleanness Grade. After the procedure, all patients were routinely monitored for 1 h.

### Outcomes

Primary outcome was procedure time. The time of examining the whole stomach was recorded, and the time for biopsy was not included. Secondary outcome was the patients’ satisfaction after the examination. A 10-point scale was used to evaluate the patients’ satisfaction (0 worst, 10 best). All the symptoms such as abdominal pain, distension, and unintended effects were also recorded.

### Statistical analysis

All the statistical analysis was performed using SPSS software. The continuous and categorical data were presented as mean (range) and percentage, respectively. The differences among three groups were tested by one-way analysis of variance (ANOVA) and chi-square if applicable. A two-tailed *P* value less than 0.05 was considered as statistically significant.

## Results

### Demographic and clinical characteristics

A total of 311 patients were included for analysis, including 110 in group 1, 92 in group 2, and 109 in group 3. There were no statistically significant differences on the demographic (age and gender) and clinical features (previous esophagogastroduodenoscopy history, indications for endoscopic examinations and positive H. pylori infection within 3 months) (all *P* values > 0.05) (Table 1).

**Table 1:** Demographic and clinical characteristics

	Group 1 (*n* = 110)	Group 2 (*n* = 92)	Group 3 (*n* = 109)	*P* value
Age, years, range	18–88	22–80	23–84	0.672
Male gender, *n* (%)	56 (50.9)	49 (53.3)	43 (39.5)	0.102
Previous history of esophagogastroduodenoscopy, *n* (%)	78 (70.9)	53 (57.6)	57 (52.3)	0.484
Indications, *n* (%)				0.331
Epigastric pain	47 (42.7)	43 (46.7)	35 (32.1)	
Dyspepsia	13 (11.8)	16 (17.4)	39 (35.8)	
Nausea	8 (7.3)	3 (3.3)	3 (2.8)	
Vomit	6 (5.5)	2 (2.2)	2 (1.8)	
Heartburn	21 (19.1)	10 (10.9)	14 (12.8)	
Surveillance	15 (13.6)	18 (19.6)	16 (14.7)	
Positive *H. pylori* infection within 3 months, *n* (%)	23 (20.9)	13 (11.8)	19 (17.4)	0.459

### Endoscopic examinations

The distribution of patients with different endoscopic and pathological diagnosis was comparable among the three groups, respectively (*P* = 0.607; *P* = 0.289) (Table 2). The mean examination time in groups 1, 2, and 3 was 8 (2–20), 6 (2–15), and 9 (2–25) min, which was not greatly different (*P* = 0.267).

**Table 2:** Endoscopic examinations

	Group 1 (*n* = 110)	Group 2 (*n* = 92)	Group 3 (*n* = 109)	*P* value
Endoscopic diagnosis, *n* (%)				
Chronic atrophic gastritis	6 (5.5)	4(4.3)	10 (9.2)	0.607
Chronic non-atrophic gastritis	89 (80.9)	67 (72.8)	93 (85.3)	
Peptic ulcer	5 (4.5)	7 (7.6)	3 (2.8)	
Polyp	9 (8.2)	12 (13.0)	3 (2.8)	
Carcinoma	1 (0.9)	2 (2.2)	0	
Biopsy, *n* (%)	43 (39.1)	59 (64.1)	55 (50.5)	0.289
Pathological diagnosis for biopsy, *n* (%)				
Chronic atrophic gastritis	4 (3.6)	2 (2.2)	4 (3.7)	
Chronic non-atrophic gastritis	22 (20.0)	18 (19.6)	36 (33.0)	
Peptic ulcer	4 (3.6)	5 (5.4)	9 (8.3)	
Adenoma	9 (8.2)	11 (12.0)	3 (2.8)	
Carcinoma	1 (0.9)	2 (2.2)	0	
Intestinal metaplasia	3 (2.7)	9 (9.8)	2 (1.8)	
High-grade intraepithelial neoplasm	0	5 (5.4)	0	
Low-grade intraepithelial neoplasm	0	7 (7.6)	1 (0.9)	
Examination time, min, median (range)	8 (2–20)	6 (2–15)	9 (2–25)	0.267
Gastric Cleanness Grade, *n* (%)				< 0.001
A	19 (17.3)	73 (79.3)	29 (26.6)	
B	19 (17.3)	12 (13.0)	41 (37.6)	
C	72 (65.5)	7 (7.6)	39 (35.8)	

However, statistically significant differences were found on gastric cleanness grade (*P* < 0.001) (Table 2). The proportion of patients with Gastric Cleanness Grade A was most in group 2 (*n* = 73, 79.3%), followed by group 3 (*n* = 29, 26.6%) and then group 1 (*n* = 19, 17.3%). Patient proportion with Gastric Cleanness Grade C was most found in group 1 (*n* = 72, 65.5%), followed by group 3 (*n* = 39, 35.8%) and group 2 (*n* = 7, 7.6%).

### Patients’ satisfaction

Satisfaction score was reported by the patients immediately and 2 h after the endoscopic examinations, but there was no statistically significant difference on the satisfaction scores [6 (3–8) vs. 6 (1–10) vs. 6 (1–9), *P* = 0.533; 10 (8–10) vs. 10 (10–10) vs. 10 (8–10), *P* = 0.463] (Table 3). For discomfort reported immediately after the endoscopy, 38 patients (34.5%) in group 1, 23 patients (25.0%) in group 2, and 29 patients (26.6%) in group 3 complained the presence of abdominal distension (*P* = 0.264). Thirteen patients (11.8%) in group 1, 8 patients (8.7%) in group 2, and 6 patients (5.5%) in group 3 had nausea (*P* = 0.252). In group 1, 24.54% patients (*n* = 27) had throat pain, which was 2.2% (*n* = 2) in group 2 and 10.8% (*n* = 10) in group 3 (*P* < 0.001) (Table 3).

**Table 3:** Satisfaction score (0–10) and discomfort after examinations

	Group 1 (*n* = 110)	Group 2 (*n* = 92)	Group 3 (*n* = 109)	*P* value
Satisfaction score, mean (range)				
Immediately after examination	6 (3–8)	6 (1–10)	6 (1–9)	0.533
2 h after examination	10 (8–10)	10 (10–10)	10 (8–10)	0.463
Discomfort reported, *n* (%)				
Abdominal distension	38 (34.5)	23 (25.0)	29 (26.6)	0.264
Nausea	13 (11.8)	8 (8.7)	6 (5.5)	0.252
Throat pain	27 (24.5)	2 (2.2)	10 (10.8)	<0.001

## Discussion

Esophagogastroduodenoscopy is the most useful tool for diagnosing and treating upper gastrointestinal mucosal lesions. At recent, great progress has been made on improving the efficiency and accuracy of the endoscopic procedures [8]. It is reported that the administration of simethicone before esophagogastroduodenoscopy can increase the detection rate of the lesions by removing the foams in the stomach [9]. However, the optimal strategy of simethicone usage has not been well clarified. Thus, in this study, we aimed to investigate the influence of different simethicone administration strategies on the gastric cleanness for esophagogastroduodenoscopy.

Sajid MS et al. ever conducted a systematic review and meta-analysis of 7 randomized controlled trials on the application of simethicone in improving the gastric mucosal visualization during esophagogastroduodenoscopy [9]. These results supported that oral simethicone administration before endoscopic examinations can improve the mucosal visualization of the stomach. Mucosal visibility score was reported in four trials, and the other three trials reported the number of patients with adequate and poor visibility [10–16]. In our present study, we defined Gastric Cleanness Grade based on the observation of the water and foam in gastric body and fundus under endoscopy, which was manifested in Fig. 2. Gastric Cleanness Grade was used to evaluate the effectiveness of simethicone in cleaning the stomach for observation under endoscopy. Our data showed that the percentage of patients with Gastric Cleanness Grade A was highest in group 2 (simethicone solution intake 31–60 min before the procedure), which was obviously higher than those in groups 1 and 3 (79.3% vs. 17.3% and 26.6%, *P* < 0.001). It was proved that simethicone solution intake 31–60 min before the procedure can obtain the best gastric visualization for endoscopic procedures, compared with pre-procedural simethicone solution intake 20–30 min and > 60 min before.

No significant differences were found on patients’ satisfaction score both immediately and 2 h after the procedure (both *P* values > 0.05), indicating that all the three simethicone intake methods were all tolerated by the patients. The incidence of symptoms reported by the patients was nearly comparable among different groups, except throat pain. Group 1 had a greatly higher percentage of patients (24.5%, *n* = 27) who had throat pain than 2.2% (*n* = 2) in group 2 and 10.8% (*n* = 10) in group 3 (*P* < 0.001). This may be partly explained by the repeated friction between endoscopy body and the pharyngeal mucosa in group 1 in order to remove the excessive water or foam in the stomach, which was consistent with the fact that the examination time in group 1 was a bit longer than that in group 2 but shorter than that in group 3 although the difference was not obviously significant [8 (2–20), 6 (2–15), and 9 (2–25) min; *P* = 0.267]. 24.5% patients in group 1 reported throat pain, which was greatly higher than those in groups 2 and 3. The difference of throat pain may be explained by the facts that individuals may have a different tolerance to the injury of throat mucosa caused by the manipulation of the endoscopy, and more patients with chronic pharyngitis were in group 1.

There were also limitations in this study. First, all the patients were included from one single center. Second, only selected demographic and clinical variables were analyzed here, and some other potential factors that may influence gastric mucosal visualization like dietary lifestyle were not investigated. Third, we did not examine the effect of simethicone combined with N-acetylcysteine, because N-acetylcysteine was not routinely used in our endoscopy center.

In summary, simethicone solution intake 31–60 min before the procedure is suggested, which can be introduced as a routine standard pre-procedural preparation for good quality esophagogastroduodenoscopy, especially for those patients with suspected malignant upper gastrointestinal lesions. Additionally, this conclusion will be further validated in a large multi-center randomized clinical trial.

## Data Availability

Not applicable.
